# Salud bucal pediátrica: sugerencias básicas durante y después de la emergencia sanitaria COVID-19 en el hogar y la clínica

**DOI:** 10.21142/2523-2754-0904-2021-091

**Published:** 2021-12-09

**Authors:** Guido Alberto Perona-Miguel de Priego, Sabina Mungi-Castañeda

**Affiliations:** 1 Departamento Académico de Estomatología del Niño y Adolescente (DAENA), Universidad Peruana Cayetano Heredia. Lima, Perú. guido.perona@upch.pe Universidad Peruana Cayetano Heredia Departamento Académico de Estomatología del Niño y Adolescente (DAENA) Universidad Peruana Cayetano Heredia Lima Peru guido.perona@upch.pe; 2 División de Odontopediatría, Universidad Científica del Sur. Lima, Perú. smungic@ucientifica.edu.pe Universidad Científica del Sur División de Odontopediatría Universidad Científica del Sur Lima Peru smungic@ucientifica.edu.pe

**Keywords:** pandemia, odontología pediátrica, salud bucal, COVID-19, emergencia sanitaria, niños, hogar, pandemics, pediatric dentistry, oral health, COVID-19, health emergency, childrens, home

## Abstract

En todos los campos de la vida humana, la pandemia ha impuesto cambios de adaptación a las profesiones para evitar la propagación de la enfermedad. En la atención odontológica se tuvo que adaptar las consultas por teleodontología y consulta de urgencias. El propósito de este trabajo es describir procedimientos prácticos para el cuidado y el tratamiento de la salud bucal pediátrica de prevención, y el manejo de los procedimientos dentales en el hogar y la clínica durante la pandemia de COVID-19.

## Abstract (en)

The COVID-19 pandemic has imposed changes of adaptation among not only the general population but also among professionals trying to prevent the spread of this disease. In dental care, teleodontology and consultations had to be adapted to avoid disease spread. The aim of this study was to describe practical procedures for the care and treatment of pediatric oral health in the prevention and management of dental procedures at home and in the clinical setting during the COVID-19 pandemic.

## INTRODUCCIÓN

Vivimos tiempos de emergencia sanitaria. La OMS declaró la epidemia de COVID-19 una Emergencia de Salud Pública de Importancia Internacional y, luego, la reconoció como una pandemia ^1^. Las vías de contagio típicas de COVID-19 son las transmisiones directas como estornudos, tos e inhalación de pequeñas partículas en el aire, así como la transmisión de contacto, es decir, contacto con las membranas mucosas orales, oculares y nasales. La COVID-19 también puede transmitirse por saliva directa o indirectamente. Los estudios indican que el 2019-nCoV puede ser transmitido por el aire en forma de aerosoles producidos durante tratamientos médicos y odontológicos ^2^.

Un área cerrada, como los consultorios dentales, donde se realizan procedimientos con producción de aerosol, se convierte en un lugar de posible propagación para el personal odontológico y sus pacientes. La fuente de las gotas puede ser nasofaríngea u orofaríngea, generalmente asociada con la saliva. Las gotas más grandes pueden producir la transmisión viral a los sujetos cercanos, mientras que las gotas más pequeñas, contaminadas con partículas virales, y que están suspendidas en el aire, pueden producir la transmisión a larga distancia, por lo que la consulta odontológica debe mantener al personal odontológico y los pacientes en un ambiente seguro ^3^.

Durante este periodo de pandemia, la gestión de la salud bucal de los niños ha cobrado una gran importancia, por lo que se deben implementar nuevos protocolos para la atención bucal que no sean considerada de emergencia. Un aspecto importante es enlazar una efectiva comunicación y educación a distancia orientadas a mantener la salud bucodental de los niños mediante la teleodontología.

La Asociación Dental Americana (ADA) propuso, el 16 de marzo de 2020, que los profesionales de la odontología postergaran todos los procedimientos electivos y que solo ofrezcan tratamientos de urgencia o emergencia, y la odontología pediátrica no es la excepción. La Asociación Americana de Odontología Pediátrica (AAPD) ha aconsejado a los dentistas que pospongan todos los procedimientos electivos, pero continúen la atención de emergencia o urgencia; asimismo, han sugerido posponer los casos de uso de anestesia general ^4^^,^^5^.

Esta pandemia debería obligarnos a analizar lo que ocurrirá en el mundo pos-COVID-19. El riesgo que plantean los procedimientos de generación de aerosoles obligó a buscar procedimientos alternativos, entre ellos las técnicas que no generan aerosoles, como el flúor diamino de plata (SDF), las restauraciones terapéuticas provisionales (ITR), las coronas Hall y el barniz de flúor.

En un mundo con COVID-19, se puede observar un retraso en el tratamiento definitivo de niños muy pequeños con caries extensa, que a menudo requieren atención en sala de operaciones. Con estos procedimientos que no generan aerosoles, se puede realizar la atención en el consultorio en lugar de usar anestesia general, que suele ser más costosa y de mayor riesgo. Por otro lado, estas técnicas no invasivas libres de aerosoles no son necesariamente definitivas y exigen una supervisión continua que no es factible para familias con transporte limitado, poca capacidad para ausentarse del trabajo u otros factores socioeconómicos ^6^.

El virus se quedará con nosotros por muchos años y está ocasionando algunos cambios en el comportamiento, como menos viajes, reducción del tabaquismo y otros hábitos dañinos para la salud, aumento del uso de vacunas, mayores ventas en línea, mayor interés por los cursos online y el distanciamiento social. El investigador de salud Peter Daszak y su equipo han estimado que, en el mundo, hay hasta 5000 cepas de coronavirus que esperan ser descubiertas en murciélagos ^7^.

Posiblemente, el futuro que nos espera sea uno en el que las pandemias y epidemias se vuelven parte de nuestra vida; por ello, la salud ocupa un lugar central en las políticas públicas. Como dentistas y, lo más importante, como profesionales de la salud, el objetivo principal debe ser preparar a la gente para esta nueva realidad. 

### Protocolos clínicos recomendados

Diferentes organismos de salud han publicado sugerencias de protocolos para la atención odontológica. Por ejemplo, la Asociación Latinoamericana de Odontopediatría (ALOP) menciona dos protocolos que se deben seguir: la teleodontología y la atención presencial de urgencia o emergencia. Amorim *et al*. la dividen en cuatro procesos: evaluación del paciente, preoperatorio, quirúrgico y posoperatorio ^8^^,^^9^.

Por un lado, todo odontólogo debe asumir que cualquier paciente puede ser asintomático, ligeramente sintomático o estar en el periodo de incubación (estimado entre 1 y 14 días), es decir, todos deben ser tratados como si estuvieran contaminados ^10^.

Los autores del presente trabajo pretenden dar a conocer sugerencias prácticas que ayuden a mantener el cuidado de la salud bucal del paciente de odontopediatría en el hogar y, cuando se requiera, en la consulta clínica. Esto requiere una gran motivación y ayuda de los padres de familia. Las fotos que presentamos han sido enviadas por los padres a nuestra teleconsulta y otras fueron tomadas por los autores en su práctica clínica ^11^.

## PROCESO PARA LA ATENCIÓN DEL PACIENTE EN LA CONSULTA ODONTOLÓGICA EN TIEMPO DE PANDEMIA

### Atención no presencial

La teleodontología es la atención a distancia que involucra el uso de telefonía, mensajes de texto, WhatsApp u otros medios digitales o plataformas virtuales, como Zoom, Teams, Google Meet, Hangouts, Skype, Facebook y Messenger.

a. Consulta asincrónica. Es aquella en la que el padre del niño contacta al odontopediatra para solicitar una opinión o evaluación. En este caso, existe un tiempo de espera entre el envío de la solicitud o requerimiento, y la respuesta.

b. Consulta sincrónica. Cuando la consulta virtual se produce en tiempo real por teléfono o, mejor, por videoconferencia, en la que se puede interactuar con el odontopediatra sobre los motivos de la consulta.

c. Consulta mixta. Cuando se realiza el seguimiento en el mediano o largo plazo de un caso específico, utilizando consultas asincrónicas y sincrónicas ^11^.

La evaluación vía teleodontología, para identificar pacientes con sospecha o potencial infección por COVID-19, se puede realizar antes de programar una cita. Las preguntas de detección iniciales más importantes son las siguientes:

• ¿Ha confirmado o sospecha haber tenido contacto con una persona con COVID-19?

• ¿Tiene historial reciente de viaje a cualquier región con elevada presencia de casos de COVID-19?

• ¿Tiene síntomas como fiebre o tos?

Una respuesta afirmativa a cualquiera de las 3 preguntas debería postergar el tratamiento dental electivo durante al menos 2 semanas ^12^.

La teleconsulta se puede ampliar con la ayuda y el compromiso de los padres para realizar controles por videollamada de control de placa bacteriana, cepillado dental, visualizar lesiones de caries o lesiones por traumatismo, y dar las indicaciones a los padres si requieren asistir o no a la consulta o el paciente puede ser controlado por videollamadas.

### Atención presencial

Si se determina necesaria la atención presencial del paciente, se deben seguir estos pasos:

a. **Programación.** Toda programación de un paciente debe hacerse con anticipación por medios en línea.

b. **Periodo preoperatorio**. El consultorio, desde su ingreso y en lugares estratégicos, debe mostrar alertas visuales (letreros y carteles) como recursos alternativos para reforzar las instrucciones de bioseguridad.

c. **Periodo operativo.** Lo primero es minimizar los objetos en las superficies, tratar de reducir la contaminación cruzada, quedando solo instrumentos y material de consumo individualizado disponible para que el paciente sea atendido.

d. **Periodo posoperatorio**. Debemos tomar la atención del control y mantenimiento del tratamiento realizado y supervisarlo, sobre todo si este implicó dolor, sangrado, control de medicación y alivio o complicación ^13^.

Todas las vías de transmisión de la COVID-19 son comunes para cualquier paciente odontológico. Los pacientes de odontología pediátrica pueden presentar riesgos adicionales como el uso de aparatos de ortodoncia removibles o fijos cuya inadecuada manipulación puede aumentar el riesgo de contaminación. Además, son de uso obligatorio los EPP para los niños muy pequeños y sus acompañantes.

## CONSIDERACIONES DE BIOSEGURIDAD PARA LA ATENCIÓN DEL PACIENTE NIÑO

### Personal

Todo el personal debe estar protegido con el uso del EPP completo: máscara quirúrgica, escudos faciales, gafas protectoras, guantes, gorra médica y trajes de protección.

Se pueden distinguir tres niveles de protección para el dentista pediátrico: 

• Protección primaria estándar para el personal en un contexto clínico: usar una gorra de trabajo desechable, mascarilla quirúrgica desechable, ropa de trabajo con bata blanca, gafas protectoras o protectores faciales, y guantes de látex o nitrilo desechables.

• Protección secundaria o avanzada: gorra desechable, máscara quirúrgica desechable, lentes, protector facial, bata de trabajo blanca con ropa aislante quirúrgica desechable o externa, y guantes de látex desechables.

• Protección terciaria o mejorada: corresponde al entrar en contacto con pacientes sospechosos o confirmados con infección por COVID-19. Aunque un paciente con sospecha de infección o confirmación no debe ser tratado, en el improbable caso de que esto suceda y el dentista pediátrico no pueda evitar el contacto cercano, se requiere ropa de protección especial. Si la ropa protectora no está disponible, se debe usar una bata de laboratorio con un traje protector desechable externo. Además, se deben emplear gorras desechables, gafas protectoras, escudos faciales, máscaras quirúrgicas desechables, guantes de látex desechables y fundas impermeables para zapatos ^14^.

Un punto importante es la forma de retirarse el EPP por parte del personal de la salud. Una revisión sistemática llegó a la conclusión de que hay más posibilidades de contagio cuando te retiras el EPP que cuando lo colocas ^15^.

### Paciente

Usar, en primer lugar, la teleodontología y, si es necesaria la consulta presencial, realizar todas las indagaciones de sus contactos y familiares cercanos. El niño debe ir acompañado por un número mínimo de personas. Además de medir la temperatura, se deben proporcionar mascarillas y vestimenta de protección médica a los pacientes y sus cuidadores ^16^.

### Enjuague bucal

Estudios recientes indican que la clorhexidina, el enjuague bucal más comúnmente utilizado en estudios dentales, no es eficaz contra el virus SARS-CoV-2. Por otro lado, se viene comentando que el cloruro de cetilpiridinio (CPC 0,07%), al parecer, baja la carga viral del paciente ^17^.

## CONSIDERACIONES BÁSICAS

### Odontopediatría preventiva en el hogar

**Figura 1:**
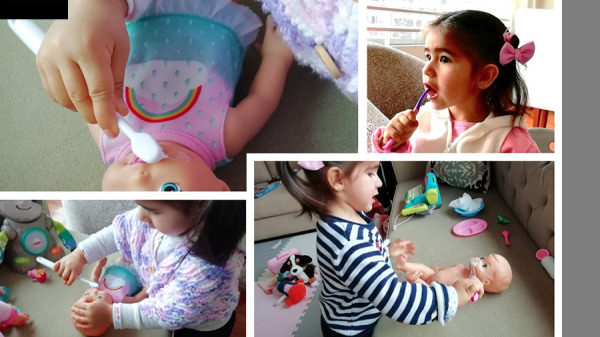
Niña jugando en casa a limpiar sus dientes, aprendiendo en tiempos de pandemia, supervisado por un odontopediatra a través de videollamada

En tiempos de pandemia, en los que el paciente pediátrico no puede asistir a la consulta en forma regular, en odontólogo debe utilizar todos los recursos *online* disponibles, como teleodontología, Facebook, Instagram o WhatsApp, para incentivar la prevención en casa por medio de avisos, folletos dirigidos tanto a los padres como a los niños. Es necesario aprovechar este tiempo en casa para iniciar los hábitos de higiene oral con los más pequeños, como el uso del cepillo a manera de juego (Figura 1).

**Figura 2:**
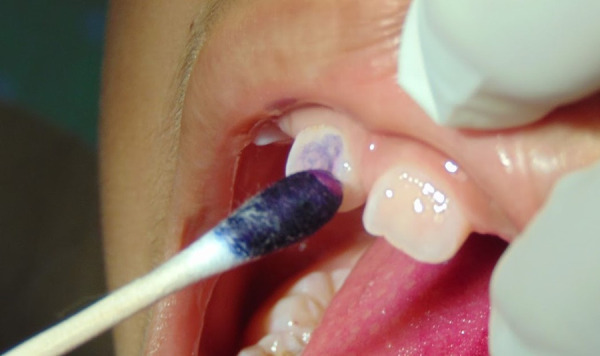
Aplicación casera de revelador de placa bacteriana mediante un hisopo, realizado por la madre y supervisado por un odontopediatra (videollamada)

También se puede hacer controles remotos de la salud y el cuidado de los dientes, como el control de placa bacteriana utilizando colorantes artificiales aplicados sobre los dientes con un hisopo y bajo la supervisión de los padres, para lo cual se envía una foto a la consulta (Figura 2).

Se recomienda a los odontopediatras enviar un kit preventivo a casa de los pacientes de riesgo que requieren controles periódicos, el cual debe incluir pasta dental con una concentración de flúor (mínimo 1000 ppm) de acuerdo con el riesgo de cada paciente, un cepillo dental adecuado para la edad del niño e hilo dental, supervisando esta actividad preventiva mediante una teleconsulta sincrónica; de esta manera no se expone al paciente durante la pandemia. Debemos mencionar que muchas de las fotos enviadas por los padres no tienen buena resolución, por lo que podemos también enseñar cómo tomar una mejor imagen de lo que desean mostrar.

### Caries

Los niños de hasta 5 años con diagnóstico de caries de aparición temprana deben ser controlados y tratados muy estrictamente desde sus etapas iniciales, ya que la evolución a un proceso infeccioso complicado puede ser difícil de tratar por teleodontología y debe ser considerado un tratamiento presencial de emergencia. Esta presencialidad es un riesgo de contaminación para los niños; por tanto, es importante el compromiso de los padres en el cuidado de sus dientes.

Durante el confinamiento por la pandemia y las clases no presenciales online, los niños pasan la mayor parte del día en casa. Igualmente, los padres se encuentran en trabajo remoto o salen a trabajar fuera de casa, los niños se quedan solos sin supervisión de consumo de alimentos cariogénicos; entonces, es muy importante la participación de los padres en saber que alimentos quedan en casa y su consumo. Es conveniente incentivar (dejando en casa) el consumo de frutas, verduras y abundante agua. ^18^

### Vigilancia y tratamiento dental remoto en el niño

**Figura 3:**
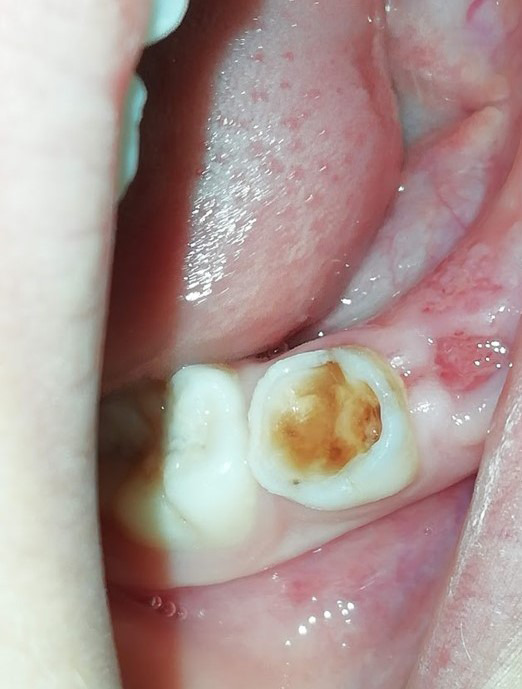
Pérdida de restauración

Durante la pandemia se pueden presentar varias situaciones clínicas que podemos manejar sin necesidad que el paciente se traslade al consultorio: dientes en proceso de tratamiento de caries y que quedaron con restauración interina, tratamiento pulpares inconclusos u observación, como eugenato o ionómero de vidrio. Debemos recomendar a los padres continuar la vigilancia de que no se pierda la restauración; de ser así y no es posible acudir a la consulta inmediatamente, deben mantenerse las cavidades limpias de restos de alimentos con un cepillado cuidadoso y enjuagatorios con agua hervida tibia. Igualmente, si hay lesiones de caries, mantener siempre la cavidad limpia con el cepillado con pasta fluorada 1,000 ppm, mínimo dos veces al día ^19^ (Figura 3).

Si se presentara la caída de una restauración temporal de un tratamiento pulpar, se deben indicar lavados de agua tibia utilizando una jeringa descartable de 20 cc sin aguja para evitar la impactación de restos alimenticios y obstruir los conductos radiculares. Estos lavados también se pueden utilizar en niños que tienen la primera molar permanente en erupción con una lengüeta de gingiva que produce retención de alimentos, adicionando enjuagatorios de clorhexidina.

**Figura 4:**
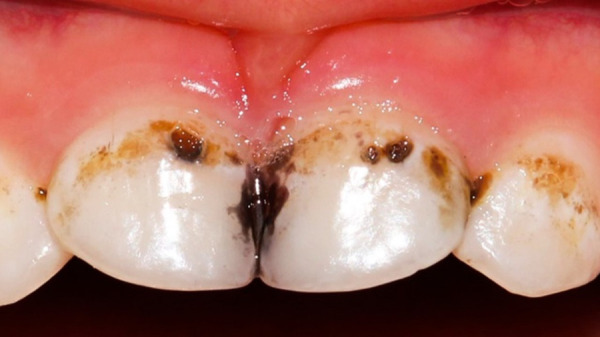
Uso del fluoruro amino de plata como control de la progresión de lesión de caries

Las lesiones de caries dental sin compromiso pulpar se pueden manejar con aplicación del fluoruro amino de plata hasta que se pueda asistir al consultorio en forma regular, y evitar en todo momento el uso de elementos rotatorios de alta velocidad con emisión de aerosol, aplicar la odontología de mínima invasión (Figura 4).

**Figura 5:**
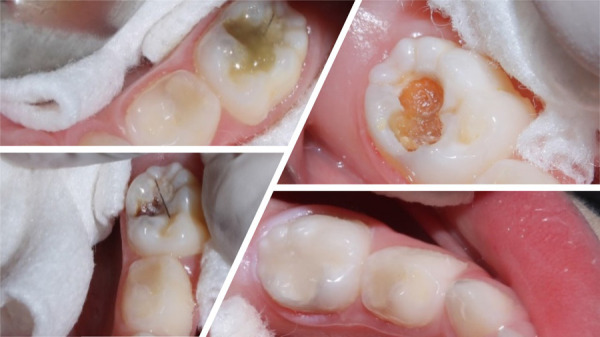
El uso de los removedores biológicos de dentina infectada evita el uso de aerosoles.

Los removedores químicos de caries son una herramienta esencial para tratar lesiones de caries y evitar el uso de aerosoles (Figura 5).

**Figura 6:**
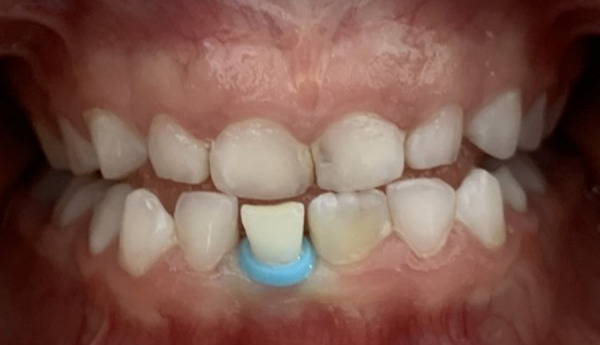
Liga separadora como ayuda para exfoliación de diente deciduo

En la etapa de recambio dental, hay muchas veces movilidad dental y retención del diente deciduo a exfoliar. Se debe instruir a los padres acerca de que no es necesario ir al consultorio, a menos que el niño tenga muchas molestias, por lo que se debe calmar e incentivar a consumir alimentos como frutas o verduras para acelerar el proceso de exfoliación. Otra manera de ayudar a exfoliar un diente deciduo será colocando una liga separadora de ortodoncia a nivel del borde cervical del diente; esta técnica ayudará a que el diente exfolie rápidamente sin necesidad de que el odontopediatra deba extraerlo (Figura 6).

### Sugerencias para el tratamiento de urgencia de ortodoncia removible

**Figura 7:**
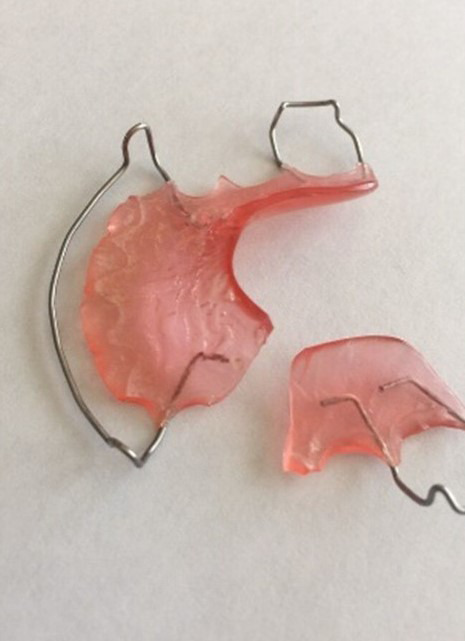
Placa removible fracturada

Toda manipulación para el retiro y la colocación de estos aparatos debe contemplar una cuidadosa higiene para evitar la contaminación. Es necesario incentivar el lavado de manos previo a la manipulación y la desinfección del aparato removible; para ello, cuando no se está usando, debe ser guardado en un recipiente de plástico y usar pastillas efervescentes de desinfección. Estos aparatos pueden sufrir roturas de los ganchos y, en ese caso, instruir al padre en el pulido del extremo con una lima de uñas nueva o una lija fina para pulido de metal; si no se pudiera usar por una fractura grande, se debe consultar si es necesaria una cita para toma de modelos nuevos (Figura 7).

**Figura 8:**
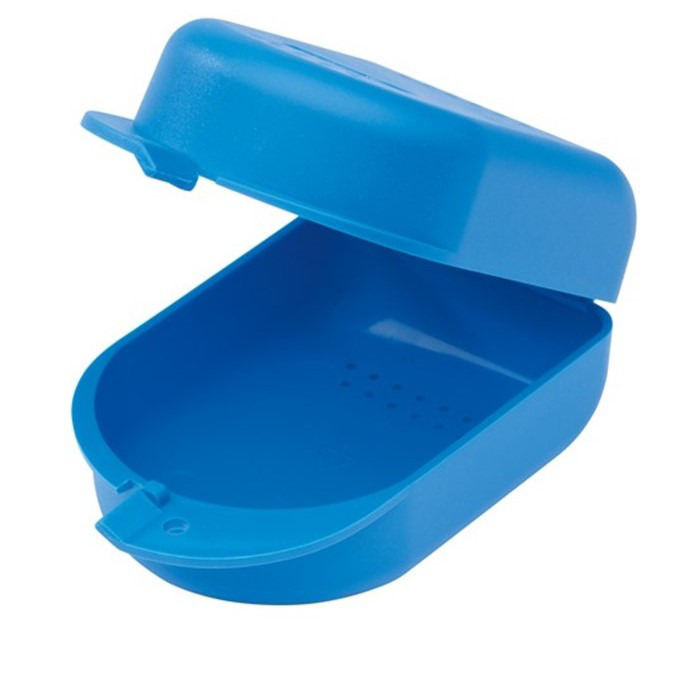
Portaretenedor de plástico

Cabe recomendar siempre que, antes de colocarlo en la boca, debe estar húmedo, no colocarlo seco. Como rutina, cada vez que se cepille los dientes, la placa de ortodoncia debe ser limpiada con pasta de dientes y enjuagarla con agua. También, como se mencionó, es importante el uso de pastillas efervescentes en un vaso con agua y dejarlo por 30 minutos. Si no se tienen a la mano las pastillas efervescentes, pueden usarse enjuagatorios bucales libres de alcohol u otros limpiadores caseros, como el bicarbonato de sodio diluido en agua. Lo importante es incentivar el buen uso de las placas removibles y mantenerlas limpias (Figura 8 y 9).

**Figura 9:**
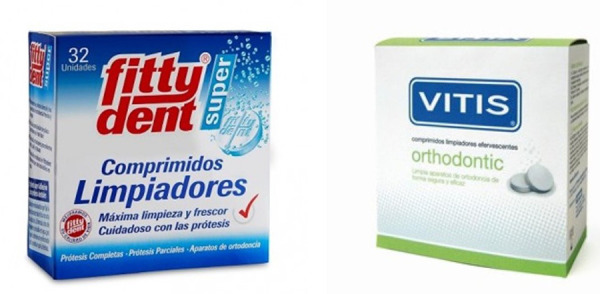
Pastillas efervecentes para limpieza de retenedores

### Tratamiento de ortodoncia fija

Los diversos aparatos que requieren activación deben ser supervisados por el odontólogo tratante, instruir a los padres que no hagan estas activaciones sin conocimiento, porque pueden crear o agravar los problemas ortodóncicos del niño. La alimentación es importante, se debe evitar ingerir alimentos como dulces, gomas o chocolates que puedan desprender los aditamentos del aparato. 

La British Orthodontic Society, a la luz de la evidencia más reciente sobre la propagación de COVID-19, ha creado un protocolo de emergencia para tratar todos los problemas de ortodoncia, excepto los más urgentes. 

La mayoría de los aparatos de ortodoncia se pueden dejar *in situ* durante algunos meses sin detrimento del paciente, si este cumple las instrucciones habituales de cuidado posterior ^20^.

Higiene bucal ejemplar. Cepillado 3 veces al día con cepillo de dientes y pasta dental de 1,000 ppm, seguido del uso de cepillo interproximal. Como complemento, uso de un enjuague bucal con flúor, por ejemplo, Fluoriguard (225 ppm), una vez al día. 

Dieta baja en azúcar. Siempre que sea posible, evitar los bocadillos de azúcares y bebidas con azúcar agregada. Las bebidas gaseosas deben evitarse en particular. 

Evitar el consumo de ciertos alimentos. Los alimentos duros, pegajosos y duros pueden desprender un *bracket*, doblarlo, deformarlo o retirar del tubo el arco.

Se recomienda que todo contacto sea primero por teleodontología o videollamada, para que el profesional o un asistente entrenado hable con el paciente o los padres a fin identificar el problema y determinar si requiere una consulta presencial o puede ser solucionado el problema en el hogar.

Se debe obtener la siguiente información:

• Un resumen del problema 

• Cualquier problema médico que pueda afectar la toma de decisiones 

• Fotos del problema tomadas con un teléfono inteligente y enviadas al consultorio para su evaluación primaria

Las preguntas básicas que deben de realizarse son las siguientes: ¿tiene dolor?, ¿cuál es el problema? Luego, se debe determinar si su puede ayudar al pacienta a resolver el problema en casa o si se trata de un problema agudo de ortodoncia que está afectando el estilo de vida de la persona y hace obligatoria su visita al consultorio.

Una vez recibida la información adecuada, se pueden seguir estas recomendaciones:

• El consejo o las instrucciones deben darse por teléfono o videollamada, siempre que sea posible.

• Se puede enviar por *delivery* al domicilio alguna solución como cera de ortodoncia, receta médica o elásticos.

• Se puede recibir en el consultorio, por *delivery*, algún aparato que requiera una reparación simple sin necesidad de la presencia del paciente en el consultorio.

• Hacer una lista de los problemas para justificar la presencia en el consultorio.

La rotura de soportes, arcos o tubos, y bandas fueron las causas más comunes de urgencias o citas de emergencia durante la etapa temprana de la pandemia ^21^.

### Casos comunes que se presentaron

**Figura 10:**
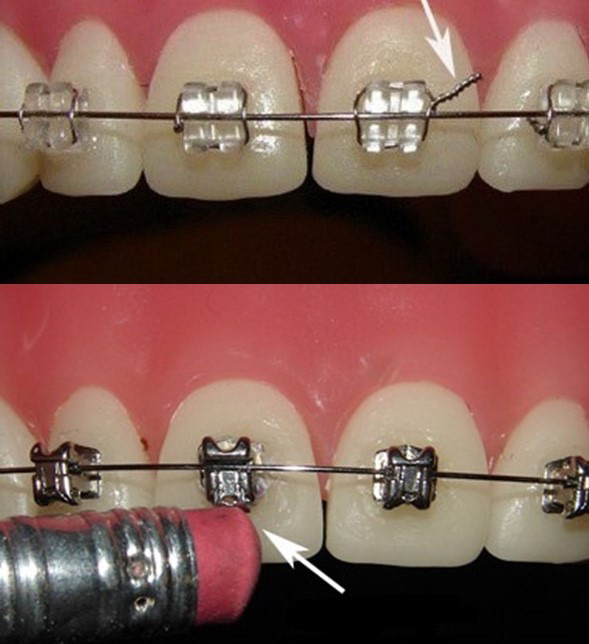
Ligadura suelta ,los padres pueden adaptarla con la punta del borrador de un lápiz.

a. **Banda suelta*.*** Si está muy suelta y causa muchas molestias orientar a los padres para que la retiren, ayudados con una gasa para evitar el tragar la misma y recortar el arco al nivel de la cara distal del último brackett con un alicate cortaúñas nuevo. Si no hay molestias y está no muy suelta puede quedar interinamente siempre y cuando observe cuidados de higiene de la zona con buena higiene oral con pasta y enjuagatorios con flúor.

b. **Arco suelto**. Si hay un alambre desprendido, puede cortase con un cortaúñas y sellar el extremo con cera. Si el alambre se ha desplazado del tubo, tratar de volverlo a colocar utilizando con cuidado una pinza para cejas. Se puede usar el extremo de goma de un lápiz para el extremo del alambre y luego consultar con fotografías al odontólogo tratante (Figuras 10 y 11).

**Figura 11:**
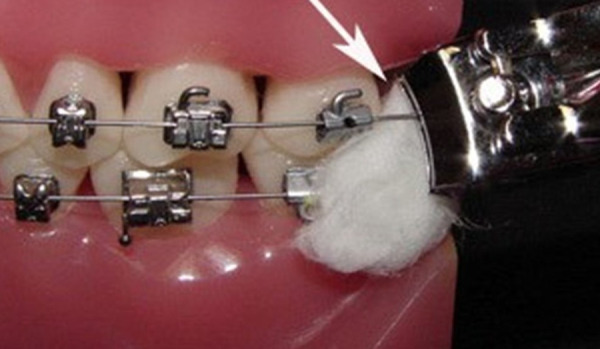
Porción distal de arco suelto, se puede cortar y colocar cera de ortodoncia.

### Casos que sí requieren atención presencial

Son todos aquellos que no se pueden solucionar en casa y que están afectando el estilo de vida del paciente. Durante el confinamiento, hubo relativamente pocas emergencias de ortodoncia, muchas de las cuales se manejaron mediante consulta telefónica; sin embargo, un porcentaje mucho menor de emergencias fueron generadas por aparatos removibles (por ejemplo, alineadores transparentes) en comparación con aparatos fijos (por ejemplo, equipos *multibracket*), lo que probablemente influyó en la decisión de la mayoría de los profesionales de optar por aparatos extraíbles a raíz de la pandemia de COVID-19 ^22^.

La situación actual de pandemia ha hecho del distanciamiento social la nueva norma para prevenir la infección cruzada. El triaje de ortodoncia personalizado para una gestión eficiente del paciente, así como la regulación de la afluencia de pacientes en el consultorio, son medidas adecuadas. Además, tiene un alcance futuro al ser incorporado en un *software* integrado capaz de automatización y videos de autoayuda para el mantenimiento en alternativas de salud en salud ^23^.

## CONSIDERACIONES QUE REQUIEREN ATENCIÓN NO PRESENCIAL Y PRESENCIAL

**Figura 12:**
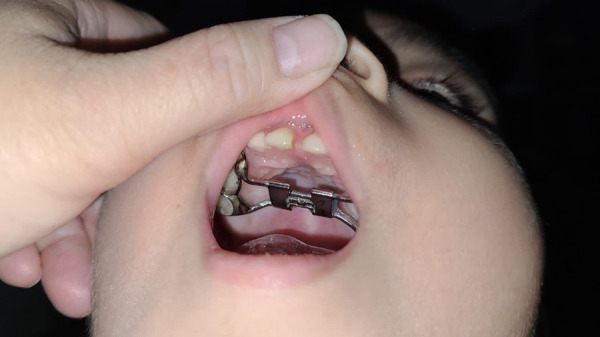
Orientación por videollamada para ajustes de aparatología de ortodoncia

**Figura 13:**
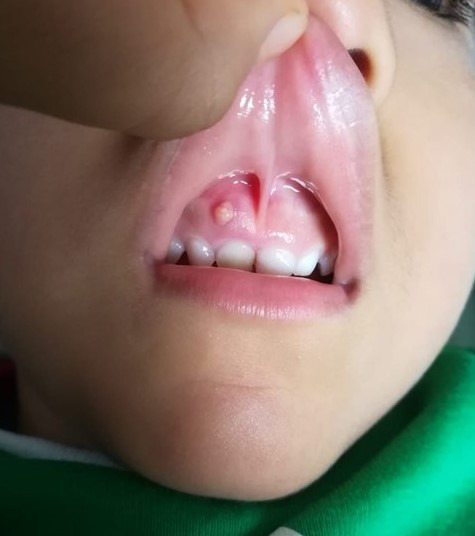
Foto enviada por madre, consulta sobre «bultito» que encontró en la boca del niño, no hay dolor ni molestias. La recomendación es solo controlarlo hasta que pueda asistir al consultorio.

**Figura 14:**
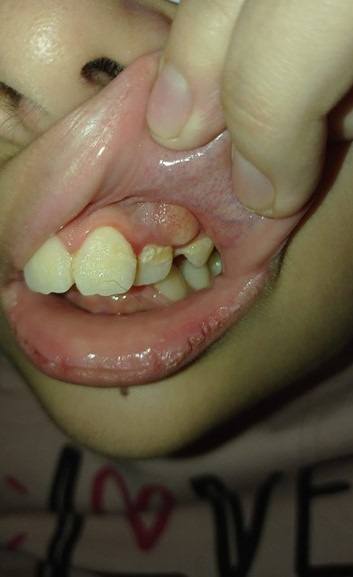
Foto enviada por madre. Consulta sobre hinchazón dura observada en la boca de su niña, no hay dolor ni molestias. La recomendación es controlar hasta que pueda asistir al consultorio. Se trata de la erupción de un premolar y hay poco espacio, por lo que va a requerir tratamiento de ortodoncia. Mejorar y controlar la higiene dental.

Orientación no presencial (Figuras 12, 13, 14)

### Urgencias/emergencias con atención presencial

Hay varias entidades clínicas que van a requerir nuestra intervención inmediata, como el trauma dental, el trauma maxilofacial, la pulpitis aguda y el absceso dental agudo. En estos casos, se debe citar al paciente y realizar el procedimiento con todas las medidas de bioseguridad cumplidas por el personal que se encuentra en el consultorio y también por el paciente y sus acompañantes, para esto se debe tener a la mano todo el material dispuesto y coordinar la cita espaciada ^24^^,^^25^.

**Figura 15:**
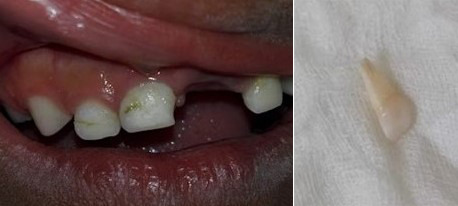
Madre envía foto de avulsión completa de diente deciduo, no requiere atención presencial, solo orientación.

Las siguientes condiciones requieren orientación o tratamiento dental pediátrico inmediato: 

• Una hinchazón propensa a comprometer la deglución o la respiración, que induce al *trismus* o diseminación a los ojos, o hinchazón oral / facial severa con pirexia (Figura 15).

**Figura 16:**
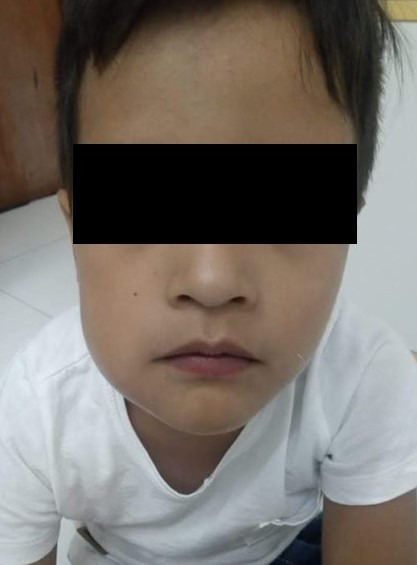
Foto enviada por madre. Niño con dolor dental y aumento de volumen de hemicara, requiere tratamiento presencial de la urgencia.

•Lesión dental traumática que puede resultar en una lesión dental permanente compleja (avulsión permanente del diente, luxación severa, fractura de la raíz y corona, fractura de corona complicada) (Figura 16).

• Lesión dental traumática de la dentición temporal (exposición a la pulpa o luxación severa tal que la movilidad del diente sea grado III)

• Sangrado gingival incontrolado, que no responde al autocuidado.

• Dolor dental severo (pulpitis irreversible) que no reacciona a los medicamentos y causa dificultad para comer o dormir.

Para la atención inmediata bajo anestesia general, los siguientes casos deben tener prioridad: 

• Niños con trauma de dentición decidua o permanente que requiere intervención, y el tratamiento con anestésicos locales o sedación consciente no es posible.

• Niños que presentan hinchazón facial (origen dental) y el tratamiento bajo anestesia local no es posible.

• Niños cuya mala salud dental está afectando o se espera que afecte a su salud.

• Niños con necesidades especiales, en los que el dolor dental produce conductas autolesivas u otras conductas disruptivas o perjudiciales ^26^.

### Lesiones traumáticas

Hemos recibido consultas vía WhatsApp de niños que sufren lesiones traumáticas dentales en casa al jugar en espacios pequeños donde hay muebles, saltar en las camas cuando no están los padres y requieren de tratamientos urgentes presenciales.

## CONCLUSIONES Y RECOMENDACIONES

• Usar primero la teleodontología.
• Utilizar la educación a distancia por redes.
• Mantener una permanente comunicación con los padres.
• Brindar atención de urgencia o emergencia presencial con estricto protocolo de bioseguridad.
• Tener ambientes de trabajo desinfectados y bien ventilados.
• Desinfectar los ambientes y equipos después de cada atención.
• Usar procedimientos de mínima intervención.
• Utilizar técnicas de odontología a cuatro manos.
• Utilizar un aspirador de alto volumen para minimizar las gotas y los aerosoles durante el funcionamiento de la turbina de alta velocidad.
• Usar el aislamiento absoluto si se van a realizar tratamientos restaurativos o pulpares.

## Fuente de financiamiento:

Autofinanciado
